# Identification of a novel marker and its associated laccase gene for regulating ear length in tropical and subtropical maize lines

**DOI:** 10.1007/s00122-024-04587-z

**Published:** 2024-04-05

**Authors:** Yaqi Bi, Fuyan Jiang, Yudong Zhang, Ziwei Li, Tianhui Kuang, Ranjan K. Shaw, Muhammad Adnan, Kunzhi Li, Xingming Fan

**Affiliations:** 1 Faculty of Life Science and Technology, Kunming University of Science and Technology, Kunming, 650093 China; 2 Institute of Food Crops, Yunnan Academy of Agricultural Sciences, Kunming, 650205 China; 3 Dehong Teachers’ College, Luxi, 678400 China

## Abstract

**Key message:**

This study revealed the identification of a novel gene, *Zm00001d042906*, that regulates maize ear length by modulating lignin synthesis and reported a molecular marker for selecting maize lines with elongated ears.

**Abstract:**

Maize ear length has garnered considerable attention due to its high correlation with yield. In this study, six maize inbred lines of significant importance in maize breeding were used as parents. The temperate maize inbred line Ye107, characterized by a short ear, was crossed with five tropical or subtropical inbred lines featuring longer ears, creating a multi-parent population displaying significant variations in ear length. Through genome-wide association studies and mutation analysis, the A/G variation at SNP_183573532 on chromosome 3 was identified as an effective site for discriminating long-ear maize. Furthermore, the associated gene *Zm00001d042906* was found to correlate with maize ear length. *Zm00001d042906* was functionally annotated as a laccase (*Lac4*), which showed activity and influenced lignin synthesis in the midsection cells of the cob, thereby regulating maize ear length. This study further reports a novel molecular marker and a new gene that can assist maize breeding programs in selecting varieties with elongated ears.

**Supplementary Information:**

The online version contains supplementary material available at 10.1007/s00122-024-04587-z.

## Introduction

Maize ear development initiates from the female spike primordia (SP). As the transition from vegetative to reproductive growth occurs, the female SP situated in the leaf axil undergoes gradual transformation into the spikelet-pair meristem, spikelet meristem, and floral meristem of the inflorescence (Vollbrecht and Schmidt 2009). Through the coordinated influence of multiple genetic networks regulating inflorescence development in maize, spikelets ultimately give rise to maize ears with varying lengths and thicknesses.

The length of maize ears plays a pivotal role in determining the number of kernels per row, exerting the most significant positive impact on yield among various maize yield components (Jia et al. 2020; Ning et al. 2021). Therefore, the increase in maize ear length (EL) directly leads to a higher yield in production (Mendes-Moreira et al. 2014; Luo et al. 2022). EL in maize is controlled by multiple genes. Currently, numerous quantitative trait loci (QTL) or candidate genes that regulate EL in maize have been identified through fine mapping or genome-wide association analysis (GWAS) (Li et al. 2011; Chen et al. 2014; Yi et al. 2019; Ning et al. 2021). Nevertheless, owing to the low size of mapping population or low marker density, further in-depth research is still required for most of these QTLs and candidate genes. Recently, a few functional genes responsible for EL have been successfully cloned. For instance, Luo et al. (2022) discovered that *YIGE1*, which encodes an unknown protein, might play a role in regulating EL by participating in sugar and auxin signaling pathways. Pei et al. (2022) observed that *EAD1* regulates ear development by modulating malate content in the apex of immature ears. Despite these discoveries, the practical application of these functional genes in maize breeding is still limited.

Tropical and subtropical maize germplasms exhibit rich genetic variations that are absent in temperate maize (Grzybowski et al. 2023). By crossing tropical and subtropical maize lines with temperate maize lines, many elite hybrid varieties have been developed (Fan et al. 2014). In this study, five tropical and subtropical maize lines and a temperate maize line that displayed notable differences in EL were used to establish a multi-parent population. Each of these six inbred lines holds significant breeding value and has served as parental lines in developing elite hybrids (Yin et al. 2022; Jiang et al. 2023; Wang et al. 2023). The objective of this study was to identify molecular markers and candidate genes closely associated with EL of maize, and investigate the regulatory mechanisms of the identified gene. This study may help lay a theoretical foundation for understanding the mechanisms underlying EL and will aid in marker-assisted breeding for EL in maize.

## Materials and methods

### Parental lines and population construction

In this study, the temperate maize inbred line Ye107, with relatively short ears (EL = 8.5 cm), was used as the common male parent. It was crossed with five tropical and subtropical maize inbred lines (YML226, TML418, TRL02, CML312, and CML373), characterized by long ears. After seven generations of selfing, a multi-parental population (MPP) comprising five distinct recombinant inbred line (RIL) populations was constructed. The MPP comprised 814 families, including120, 216, 151, 151, and 176 families in RIL_YML226, RIL_TML418, RIL_TRL02, RIL_CML312, and RIL_CML373 populations, respectively. The pedigrees, ecological types and heterotic groups of the six parental lines are presented in Table 1.

**Table 1:** Parental lines utilized in constructing the multi-parental population (MPP)

Parental lines	Pedigree	Heterotic group	Ecotypes	Ear length (cm)
Ye107	Selected from US hybrid DeKalb XL80	Reid	Temperate	8.5
YML226	(CML226/(CATETO DC1276/7619))F2-25-1-B-1-2-1-1-2(DH)	Non-Reid	Tropical	13.4
TML418	Derived from Thailand Monsanto hybrid	Non-Reid	Subtropical	16.8
TRL02	Derived from US hybrid	Non-Reid	Subtropical	13.9
CML312	S89500-F2-2-2-1-1-B	Non-Reid	Tropical	14.7
CML373	P43SR-4-1-1-2-1-B-8-1-B	Non-Reid	Tropical	13.1

### Field trial and phenotypic data analysis

The MPP was planted in Dehong (98°58' E, 24°43' N), Jinghong (100°78′ E, 22°00′ N) and Baoshan (99° 16′ E, 25°11′ N) in Yunnan Province, China, in 2021 and 2022. A complete randomized block design was implemented at each location with two replications. The field experiment was conducted with a row length of 3 m, inter-row space of 0.70 m, 14 plants per row, and two rows per plot. The plant spacing in a row was set at 0.25 m, resulting in a plant density of approximately 62,112 plants per hectare. Field management was conducted according to local standard agronomic practices. After 65 days of pollination, the EL of ten plants situated in the middle of each row was documented to determine the average EL. Phenotypic data were analyzed using R software (v3.2.2) to assess normal distribution. Broad-sense heritability was computed using the lme4 (v1.1–31.1) software package, using the formula h = Vg/(Vg + (Ve/L)), where Vg represents the genetic variance, Ve is the residual variance, and L is the number of environments. Multifactor analysis of variance (ANOVA) was performed using the Statsmodels (v 0.14.1) library in Python.

### Genotyping and principal component analysis

Genomic DNA was extracted from maize seedling leaves using a modified CTAB method (Allen et al. 2006). The DNA concentration was quantified using Qubit and then diluted to 20 ng/μl for library construction, following the protocol of Poland et al. (2011). The six parental lines and five F_1_ hybrids were sequenced by whole-genome re-sequenceing (WGS) using Illumina HiSeq TM platform (Illumina, SanDiego, CA, USA), while the 814 families of MPP were sequenced by genotyping-by-sequencing (GBS) on pair-end 150 Illumina Novaseq platform (Illumina, SanDiego, CA, USA). Clean reads were obtained after quality control, and aligned to the maize reference genome Zm_B73_REFERENCE_GRAMENE_4.0 using BWA software. Single nucleotide polymorphisms (SNPs) were identified using the Genome Analysis Toolkit (McKenna et al. 2010; Jiao et al. 2017). The genetic relationship matrix (GRM) was obtained using GCTA software (1.94.1) with the -make-grm parameter, and the first three principal components were extracted for principal component analysis (PCA) using the -pca3 option.

### GWAS and haplotype analysis

The MPP was used for conducting GWAS to identify candidate functional genes regulating EL. The mixed linear model of the EMMAX (v intel64-20,120,205) software package was employed for GWAS analysis, and the significant threshold was determined using Plink (v 1.9) to identify SNPs associated with EL of maize (Jiang et al. 2023). Linkage disequilibrium (LD) analysis was performed using PopLD to determine the distance at which LD decayed by half in the population. Genes associated with EL were subsequently identified within the 50 kb upstream and downstream of the significantly associated SNPs (Jiang et al. 2023; Xu et al. 2024). Haplotype analysis of functional genes was carried out using the Haplotype Caller-based method (Li et al. 2022).

### Comparative expression analysis of functional gene in the parental lines

Ears were harvested from the six parental lines at the 9-leaf stage (V9), tasseling stage, and silking stage, with subsequent recording of EL. The top part (10% of the EL) and middle part (50% of the EL) of the cobs were designated as the tip and midsections, respectively. These sections were sampled to assess the relative expression (RE) levels of the identified gene. RE levels were quantified with three replicates through fluorescence quantitative RT-PCR (qRT-PCR) using the Tiangen SuperReal PreMix Plus (SYBR Green) kit (Tiangen, Beijing). The total reaction volume for each sample was 20 μL. The gene expression was carried out using the following pair of primers: F-5'ATGGCGATCTCCTCTGCTCTT and R-5'TGCCTCGTGATGCCTTGC. Maize *Actin1* gene was used as internal control for the normalization of gene expression (Li et al. 2019). The qRT-PCR was conducted using the following program: initial pre-denaturation at 95 °C for 3 min, followed by denaturation at 95 °C for 20 s, and annealing and extension at 60 °C for 30 s. This cycle was repeated for 40 cycles. Fluorescence signals were captured during the annealing and extension steps.

### Variation analysis of functional gene in TML418 and the hybrids

SNP calling was performed by aligning the re-sequencing reads of the five F_1_ hybrids used in developing the MPP to the Zm-B73-REFERENCE-GRAMENE-4.0 reference genome. This process was accomplished using GATK4 (v4.2) software in Haplotyper Caller mode. The integrated vcf file was obtained for annotation using ANNOVAR. Subsequently, genotypic files and amino acid variation files for the corresponding segments were extracted. The position information of functional genes was determined according to the annotation file (Zm-B73-REFERENCE-GRAMENE-4.0_Zm00001d.1.gff3). The amino acid sequence information of the functional gene was extracted from the Zm-B73-REFERENCE-GRAMENE-4.0_Zm00001d.1.pep file. The variant information of SNPs and amino acids was aligned using DNAMAN. The conserved domain was identified using the NCBI Conserved Domain Search Tool (https://www.ncbi.nlm.nih.gov/Structure/bwrpsb/bwrpsb.cgi), and motif prediction for the functional gene was performed using MEME online software (https://meme-suite.org/meme/tools/meme).

### Homology analysis of the functional gene

To determine the function of the identified gene, homologous gene sequences in *Arabidopsis* and *Oryza* *sativa* were analyzed using BLAST (v2.2.26) with parameters “-e 1e-07 -a 15 -F F -m 8”. Gene family identification for the functional genes was performed using the OrthoMCL process, available at http://orthomcl.org/orthomcl/. A neighbor-joining tree was constructed using Treebest software (v1.9.2) with 1000 bootstrap replicates. The resulting neighbor-joining tree was visualized, annotated, and adjusted using iTOL (Interactive Tree of Life, available at https://itol.embl.de/).

### Measurements of lignin content and enzyme activity

Lignin content, laccase (LAC) activity, and peroxidase (POD) activity had been measured using the same samples as for RE quantification. Lignin content was measured following a modified method reported by Chen et al. (2018) with three replicates for each sample. The ear samples were weighted, and 100 mg of tissues were placed in a centrifuge tube. Subsequently, 4 mL of 80% (v/v) ethanol was added, allowing it to stand for 2 h. The mixture was then centrifuged at 12,000 rpm for 10 min to settle the precipitate. The precipitate was dissolved by immersing it in 4 mL of 80% (v/v) ethanol in a water bath for 2 h at 80 °C. Following this, the solution was centrifuged at 12,000 rpm for 10 min to collect the precipitate. Then, 4 mL of chloroform was added to the solution at 62 °C in a water bath for 1 h. The mixture was then subjected to centrifugation as previously described, to collect the precipitate. The extraction process was repeated by employing 80% ethanol and chloroform. After each extraction, the precipitate was subjected to drying in an oven at 60 °C for 2 days. The dry matter was treated with 3.6 mL of 25% (v/v) acetyl bromide solution in 2.7% (v/v) perchloric acid in acetic acid. This mixture was then subjected to water bath at 70 °C for 1 h. In each sample, 2 mL of a solution comprising of 17.24% (v/v) 2 mol/L sodium hydroxide and 82.76% (v/v) acetic acid was added. Subsequently, 0.15 ml of 7.5 mol/L hydroxylamine hydrochloride was added to terminate the reaction. The absorbance at A280 was measured using a spectrophotometer, and the lignin content was determined by referencing a standard curve.

The activities of LAC and POD in the samples were measured using Laccase Activity Assay Kit (BC1630) and Peroxidase Activity Assay Kit (BC0090), respectively, obtained from Solarbio Company (Beijing, China). Enzyme activities were calculated based on protein concentration. The protein concentration was determined using the Bradford Protein Assay Kit (PC0010) procured from Solarbio Company (Beijing, China) using a microplate reader. Three replicates were used for each sample. Significant differences between different stages of each sampling section and between the tip and midsection parts of each sampling stage were analyzed using GraphPad Prism (v9.0). Pearson correlation analysis of EL, RE, lignin content, and enzyme activity were performed using SPSS software (v22.0).

## Results

### Phenotypic data analysis

**Fig. 1:**
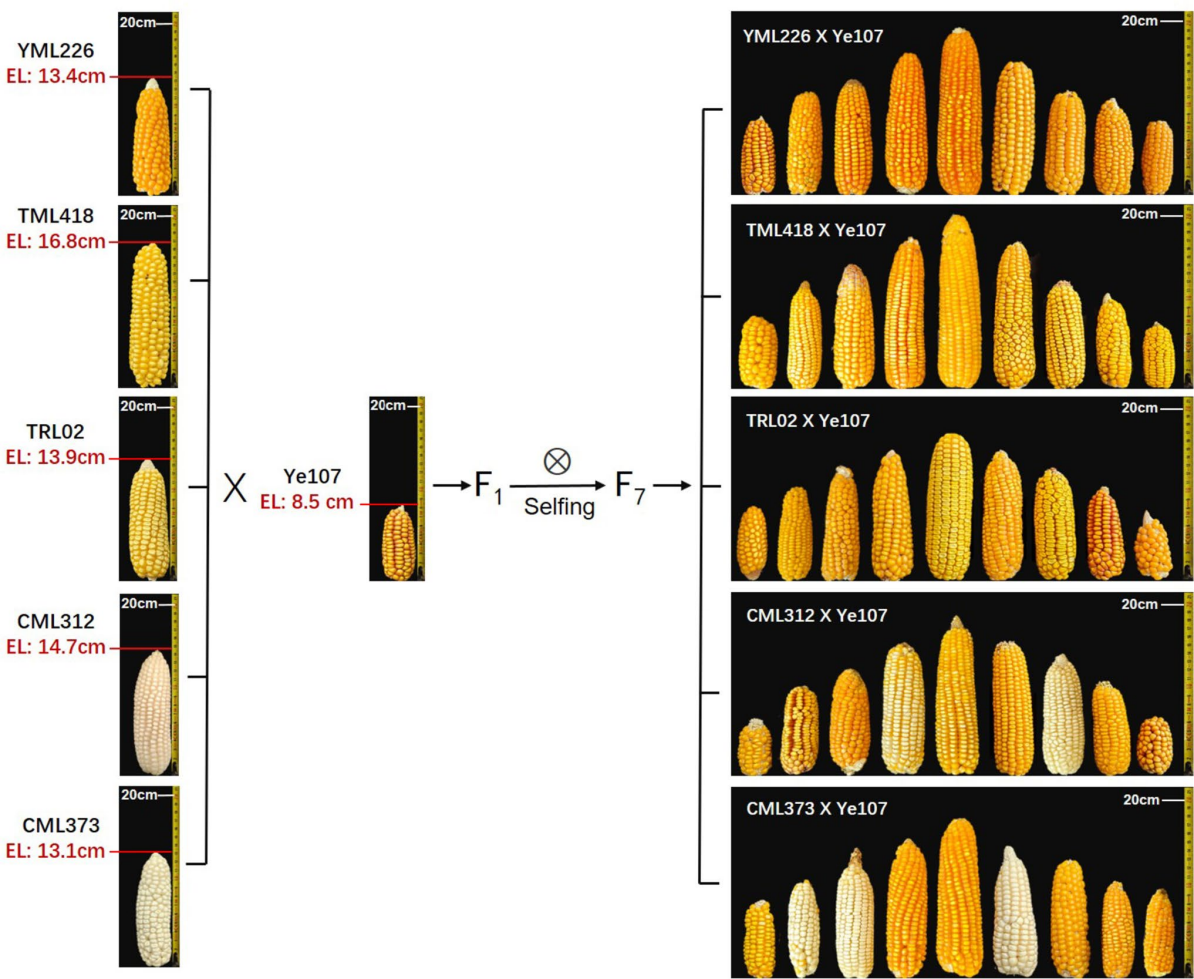
The MPP with a significant difference in EL established through the crossbreeding of a short-ear parent Ye107, with five long-ear subtropical lines

**Fig. 2:**
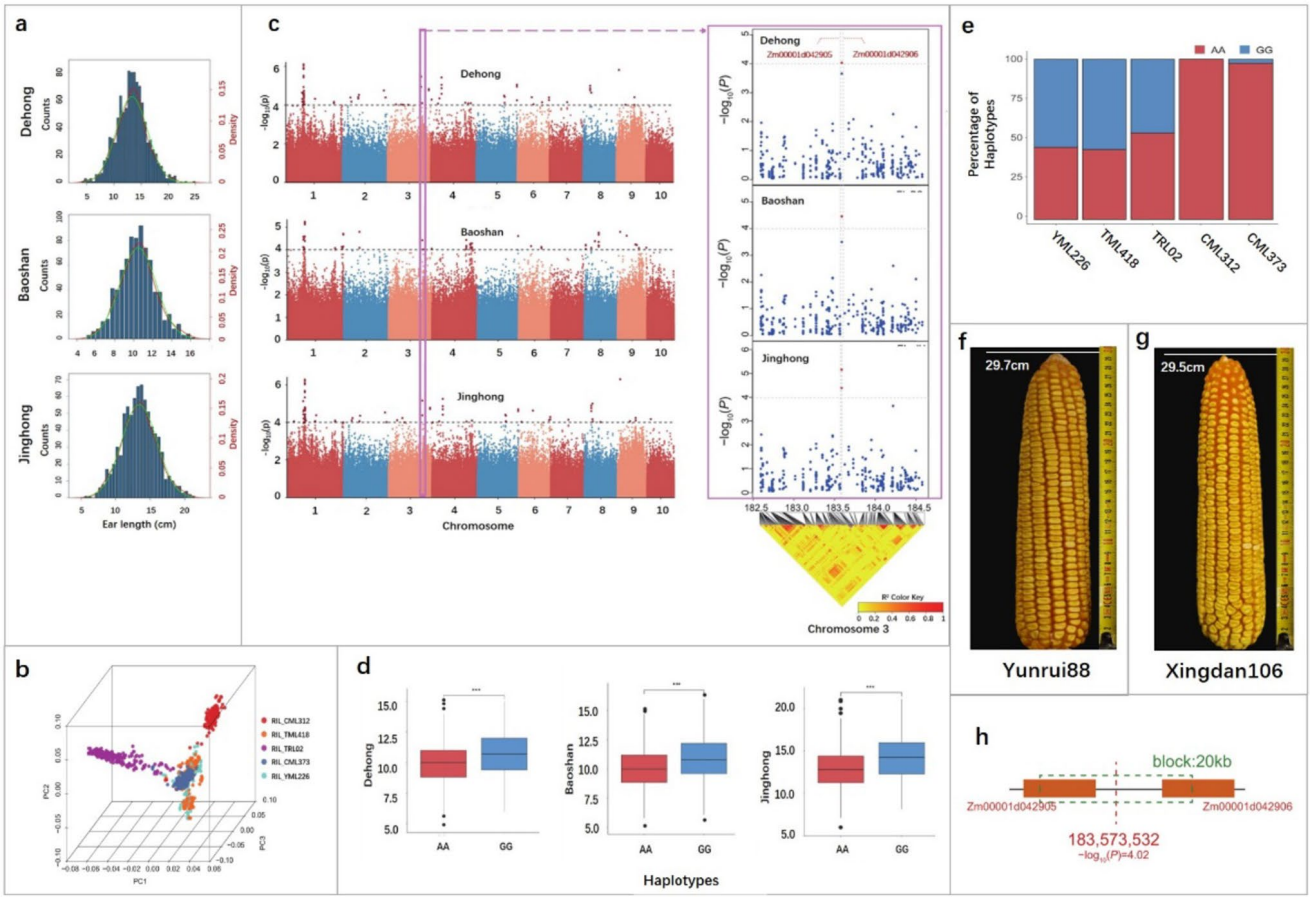
Analysis of phenotypic and genotypic data. **a** Frequency distributions of EL of the MPP at three locations. **b** Principal component analysis of MPP. Red, orange, purple, blue and green dots represent the families of RIL_CML312, RIL_TML418, RIL_TRL02, RIL_CML373 and RIL_YML226, respectively. **c** GWAS results of the MPP at three locations. **d** Significant differences in EL between two haplotypes (AA, GG) at three locations, with ***indicating *P* < 0.0001. **e** The proportions of the two haplotypes observed in five RIL subpopulations. **f** Maize hybrid Yunrui88 resulting from the cross TRL02 × Ye107. **g** Hybrid Xingdan106 obtained from the cross TML418 × Ye107 and **h** the linkage between genes *Zm00001d042905* and *Zm00001d042906* (colour figure online)

Phenotypic data revealed significant differences in EL among various RIL subpopulations within the MPP (Fig. 1). Normalization analysis of the phenotypic data from Dehong, Baoshan, and Jinghong demonstrated that EL in the MPP followed a normal distribution at all three locations (Fig. 2a). The normal distribution of phenotypic data suggests that the MPP is suitable for GWAS, as illustrated in Fig. 2a. The broad-sense heritability of EL in MPP was 0.93. ANOVA indicated significant differences in EL among the RIL subpopulations of the MPP across the three locations (*P* < 0.01). However, no significant differences were observed between the location and RIL (Table 2). Correlation analysis revealed Spearman correlations ranging from 0.74–0.98 (*P* < 0.05) among the RIL subpopulations across the three locations.

**Table 2:** Analysis of variance (ANOVA) for the EL of the MPP at three locations in Yunnan province

Source of variation	df	Sum of squares	Mean squares	F value	Pr^†^ > F
Location (loc)	2	2994.08	1497.04	218.24	< 0.0001
RIL population (RIL)	4	734.15	183.57	26.76	< 0.0001
Loc × RIL	8	102.93	12.87	1.88	0.0598
Error	1697	11,640.72	6.86		

^†^Pr, Probability

### SNP genotype and PCA analysis

After filtration, a total of 594,577 effective SNPs from GBS were identified and distributed across 10 chromosomes of maize. The highest number of SNPs was identified on Chr1 (83,599 SNPs), whereas the lowest was observed on Chr10 (43,750 SNPs). Across the genome, the SNP density was 281.11 per Mb, with the highest marker density on Chr4 (310.47 per Mb) and the lowest on Chr5 (260.27 per Mb) (Supplementary Table 1). PCA revealed that the five RIL subpopulations were generally independent of each other, although some families clustered with other RILs due to the common parent Ye107 used in MPP development (Fig. 2b).

### GWAS and identification of functional genes

GWAS analysis of maize EL was conducted using 594,577 effective SNPs. The results showed that SNPs significantly correlated with EL could be identified on all 10 maize chromosomes across various environments (Fig. 2c). By assessing the annotations of each gene where significantly associated SNPs were located, an SNP at 183,573,532 bp on chromosome 3, associated with maize EL, was consistently identified across multiple environments. Haplotype analysis of the five RILs of MPP revealed two haplotypes, AA and GG, at this particular location. Analysis of haplotypes in MPP from Dehong, Baoshan, and Jinghong locations demonstrated that haplotype GG led to a significant increase in maize EL compared with haplotype AA (Fig. 2d). Further analysis of the five RIL subpopulations revealed that RIL_TML418, derived from the parent with the longest EL, TML418, exhibited the highest proportion of the GG haplotype (Fig. 2e). This observation suggests that the GG haplotype has a positive effect on increasing maize EL.

Genotyping data for the five F_1_ hybrids of MPP for SNP_183573532 were also obtained. The results indicated that the F_1_ hybrids of TRL02 × Ye107, TML418 × Ye107, and YML226 × Ye107 had the heterozygous genotype AG, while the F_1_ hybrids of CML312 × Ye107 and CML373 × Ye107 had the homozygous AA genotype at this location. The results revealed that the long-ear parental lines TRL02, TML418, and YML226 carried the A to G mutation at SNP_183573532, indicating a strong impact of this mutation on the selection of long-ear maize. Notably, by crossing TRL02 with Ye107, an elite hybrid variety named Yunrui88 (Fig. 2f) was developed. In the demonstration of a 100 mu (1 mu = 666.67 m^2^) areas in Kunming, the average yield reached 1134.4 kg per mu, which set a record of the highest yield in 100 mu area in tropical and subtropical regions. Consequently, Yunrui88 was cultivated across an extensive area of 350,000 hectares from 2012 to 2014, and was recognized as a leading variety by the Ministry of Agriculture of China in both 2014 and 2015 (Jiang et al. 2023). Another notable hybrid, Xingdan106 (Fig. 2g), obtained from the cross TML418 × Ye107, achieved an impressive yield of 1078.1 kg per mu in demonstration areas in Southwest China in 2022 (Wang et al. 2023). The heterozygous AG at SNP_183573532 in the two elite hybrids strongly suggests that the A to G mutation at SNP_183573532 is an effective marker for selecting long-ear maize, especially in hybrids.

A range of 50 kb upstream and downstream of SNP_183573532 was explored to identify functional genes associated with EL, and two genes, *Zm00001d042905* and *Zm00001d042906*, were identified (Fig. 2c). The interval between *Zm00001d042905* and *Zm00001d042906* was 7.8 kb (Fig. 2h), which showed that the two genes were closely linked, as the interval was significantly lesser than the average distance between gene blocks (Li et al. 2022). The distance between the upstream and downstream adjacent genes of *Zm00001d042905* and *Zm00001d042906* was found to exceed 50 kb. Hence, our observations suggest that *Zm00001d042905* and *Zm00001d042906* are two closely linked genes in the target region associated with maize EL.

### Analysis of relative expression levels of *Zm00001d042906*

**Fig. 3:**
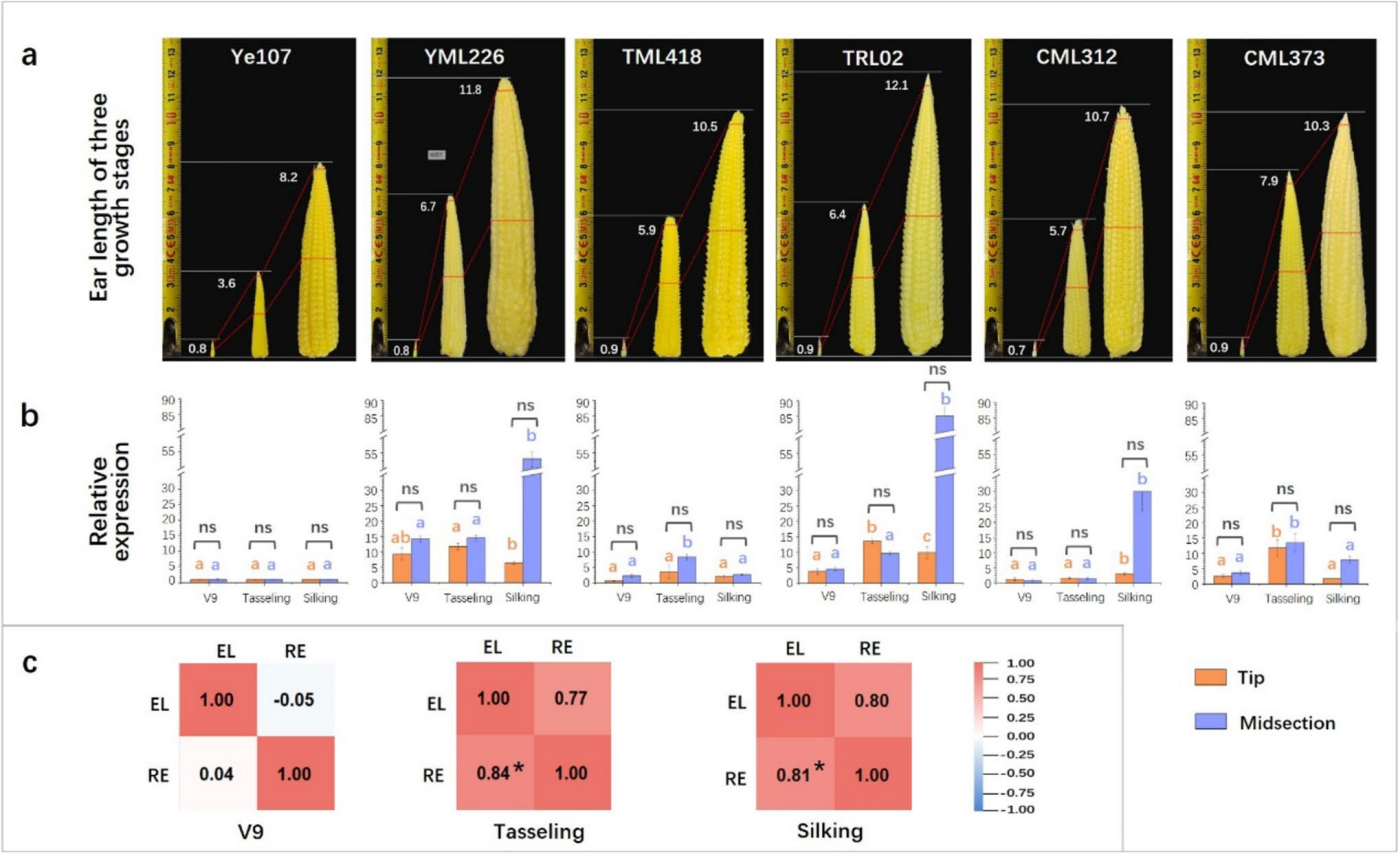
**a** EL of each parental lines at V9, tasseling and silking stages, with red dotted lines indicating the tip and midsection parts for sampling. **b** The RE of *Zm00001d042906* in the tip and midsection tissues of six parental lines at V9 stage, tasseling and silking stages. From left to right are Ye107, YML226, TML418, TRL02, CML312 and CML373, respectively. Orange bars represent the tissue collected from the tip of the cob, and purple bars represent the midsection of the cob. The orange and purple lowercase letters above the corresponding columns represent the significance level at *P* < 0.001 of the tip and midsection tissues at three sampling stages. The symbols ns, ** and *** indicate no significance, significance at *P* < 0.005 and at *P* < 0.001, respectively, between the tip and midsection tissues. **c** Correlation analysis between EL and RE for the gene *Zm00001d042906*. The data above diagonal reports correlation coefficients for tip section of ears, while data under diagonal reports correlation coefficients for the midsection of ears. The * indicates significance at *P* < 0.05. From left to right are V9, tasseling and silking stages, respectively (colour figure online)

To investigate whether gene expression differed between long and short ears, qRT-PCR was conducted by sampling the six parental lines. By calculating the distances between SNP_183573532 and the promoters of the two candidate genes, the downstream gene *Zm00001d042906* was chosen because it was closer to the SNP (the distances between SNP_183573532 and the promoter start of *Zm00001d042905* and *Zm00001d042906* were 7522 and 2495 bp, respectively). Throughout the V9, tasseling, and silking stages of maize growth, the relative expression (RE) of *Zm00001d042906* in the tip and midsection tissues of the ear cob were measured (Fig. 3a). The findings revealed that at the V9 stage, the highest expression of *Zm00001d042906* was observed in YML226, when considering the short-ear parental line Ye107 as a reference. Additionally, the RE of *Zm00001d042906* in the midsection of the ear was slightly higher than that in the tip tissue, except for CML312. At the tasseling stage, the RE of *Zm00001d042906* exhibited a significant increase from the V9 stage in TML418, TRL02 and CML373 parental lines. Moreover, the RE in the midsection remained higher than that in the tip, except for TRL02, although the differences were not significant. At the silking stage, the RE of *Zm00001d042906* varied among different parental lines. Notably, in TRL02, YML226, and CML312, the RE in midsection tissues greatly increased, reaching 85.08, 53.02, and 30.06 times higher compared to Ye107, respectively (Fig. 3b, Supplementary Table 2). However, no significant differences were observed between the two sections (Fig. 3b). This may be attributed to the relatively small sample size. Correlation coefficients were calculated between EL and RE of *Zm00001d042906* of the two ear sections during the V9, tasseling, and silking stages. The results showed that the correlations between EL and RE in the midsection were *r* = 0.04, 0.84 (*P* < 0.05), and 0.81 (*P* < 0.05) at the V9, tasseling, and silking stages, respectively (Fig. 3c). Furthermore, no correlation was observed between the EL and RE at the tip during these stages (Fig. 3c). These findings strongly indicate that the increased RE of *Zm00001d042906* in the midsection of the cob at the tasseling and silking stages is directly associated with maize EL, which confirmed role of this gene in controlling EL. Notably, the RE of *Zm00001d042906* in TML418 was consistently lower than that of the other long-ear parents across all the three stages.

**Fig. 4:**
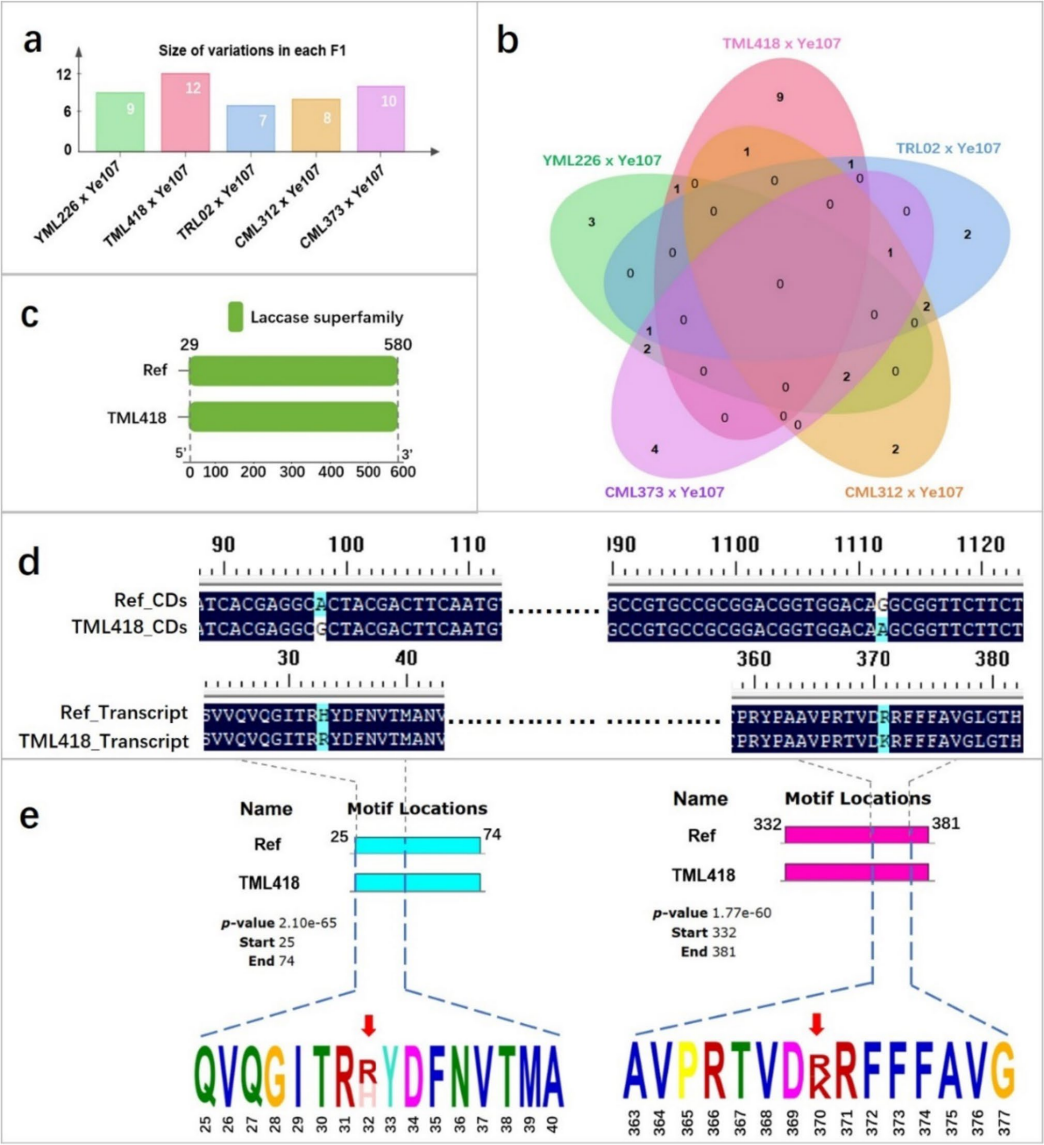
Changes of domain and motifs by the non-synonymous SNP in the coding region of *Zm00001d042906* in TML418. **a** The number of SNP variations in coding region of *Zm00001d042906* of five F_1_ hybrids; **b** the number of unique heterozygous SNPs in coding region of *Zm00001d042906* in five F_1_ hybrids; **c** conserved domain of *Zm00001d042906*; **d** change of amino acid by the non-synonymous SNP; **e** changes of motifs by the alteration of amino acid at 32 and 370 positions

Among the five long-ear parental lines used to develop the MPP, TML418 exhibited the longest EL after maturity (Fig. 1, Table 1). However, the RE of *Zm00001d042906* in TML418 was relatively low. To investigate whether the gene has mutated in TML418, five F_1_ hybrids resulting from crosses between Ye107 and the five long-ear parents were analyzed. The F_1_ hybrid of TML418 × Ye107 exhibited the highest number of SNP variations in the coding region of *Zm00001d042906* (Fig. 4a, Supplementary Table 3). Among these, nine SNPs were unique to the F_1_ hybrid of TML418 × Ye107 (Fig. 4b). A conserved domain of the laccase family was identified in the amino acid sequence spanning positions 29–580 in the coding region of *Zm00001d042906* (Fig. 4c). Further investigation revealed two non-synonymous SNPs in the nucleotide sequence of the conserved domain of *Zm00001d042906*. These two synonymous SNPs alter the amino acid sequence at positions 33 and 371 (Fig. 4d), resulting in the alteration of motifs (Fig. 4e). Therefore, it is speculated that the function of *Zm00001d042906* in TML418 was altered by the mutation of SNPs, subsequently affecting its expression.

### Prediction of the functions of genes regulating ear length

**Fig. 5:**
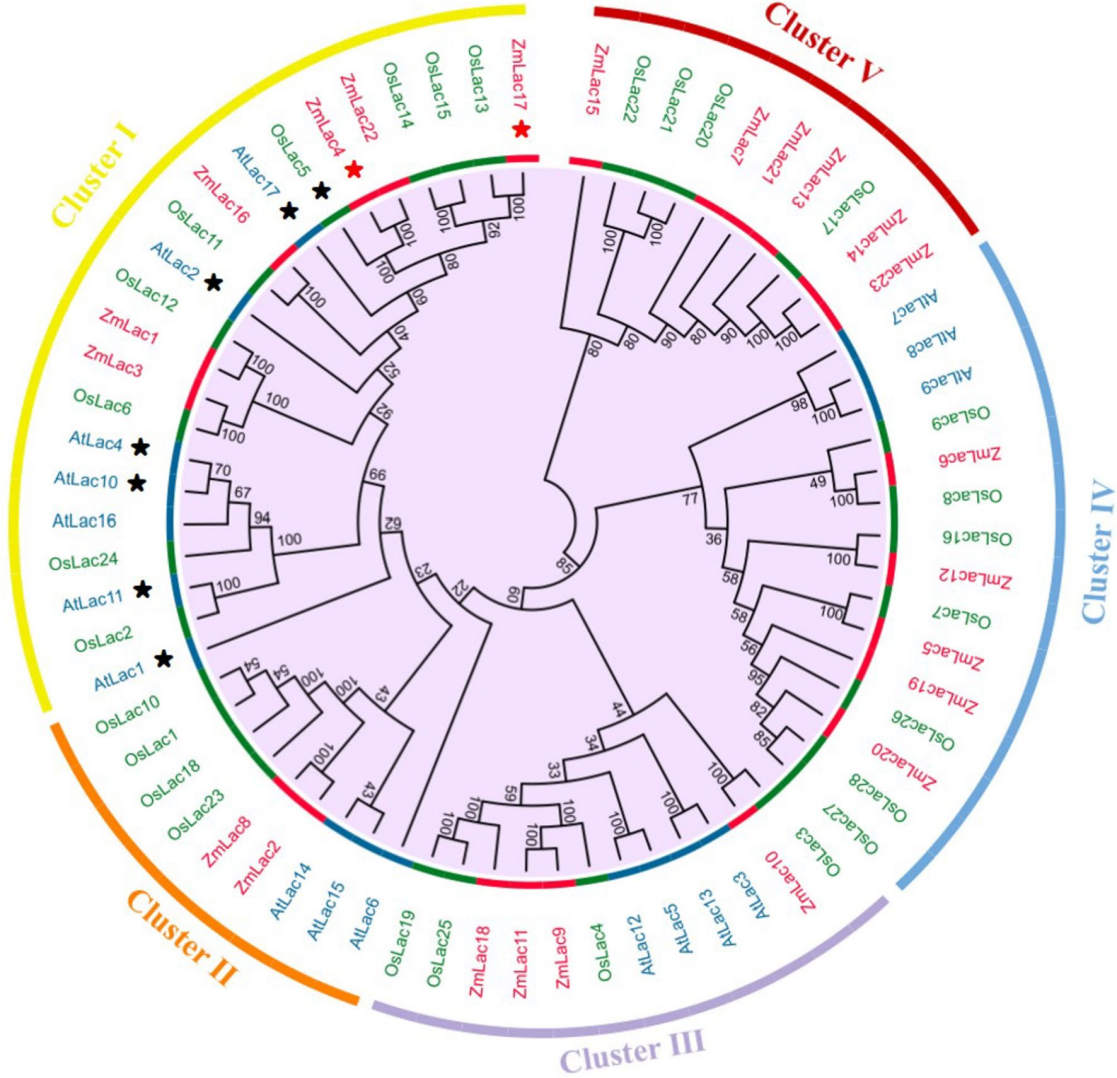
Phylogenetic tree of the reported laccases of *Z. mays* (*ZmLac*)*, O. sativa* (*OsLac*) an*d A. thaliana* laccases (*AtLac*). Red, green and blue label represents laccase of *ZmLac*, *OsLac* and *AtLac*, respectively. The seven laccases marked with black stars have been previously reported to participate in lignin synthesis. The two laccases marked with red stars represent the functional genes identified in this study (colour figure online)

According to gene annotation, *Zm00001d042906* was identified as a laccase gene known as *Lac4*. Laccase genes play roles in various metabolic activities, but the function of *Lac4* in maize remains unknown. To predict the function of *Zm00001d042906*, a homology analysis targeting *Zm00001d042906* was conducted. This analysis revealed that the homology of *Zm00001d042906* is highly conserved in both *Oryza* *sativa* and *Arabidopsis thaliana* and is associated with the laccase gene (Table 3). A phylogenetic tree was constructed to elucidate the function of *Zm00001d042906*, incorporating 23 reported maize laccase genes, 28 *Oryza* *sativa* laccase genes, and 17 *Arabidopsis* laccase genes (Supplementary Table 4). The results revealed that all laccases could be categorized into five clusters. Laccases, such as *OsLac5*, *AtLac4*, *AtLac11*, and *AtLac17*, which are known to be involved in lignin synthesis, were predominantly grouped in Cluster 1 (Lafayette et al. 1999; Zhao et al. 2013). *AtLac3*, *AtLac5*, and *AtLac13* in cluster III exhibited close associations with the formation of Casparian strips (Rojas-Murcia et al. 2020) (Fig. 5). *Zm00001d042906* (*ZmLac4*) *and Zm00001d042905* (*ZmLac17*), identified in this study, clustered with laccase genes known to regulate lignin synthesis (Fig. 5). Hence, it can be inferred that the genes identified in this study are involved in lignin synthesis in maize. Specifically, *Zm00001d042906* (*ZmLac4*) exhibited a closer relationship with previously reported *OsLac5*, which is involved in lignin synthesis (Fig. 5). Consequently, the correlations between the lignin content, LAC activity, POD activity, and RE of *Zm00001d042906* were investigated.

**Table 3:** Homology analysis of *Zm00001d042906*

Gene ID	Species	Description	Location (Chr: start–end)	Protein length	Target identity (%)	Query identity (%)
LOC4327500	*O. sativa*	Laccase-4-like	1:36,165,917–36,168,951	579	100	100
LOC107277101	*O. sativa*	Putative laccase-16	1:15,648,465–15,661,413	584	100	100
LOC4327501	*O. sativa*	Laccase-13-like	1:36,177,288–36,180,140	577	100	100
LAC16	*A. thaliana*	Laccase 16	5:23,789,362–23,792,382	567	100	100
LAC1	*A. thaliana*	Laccase 1	1:6,238,838–6,241,510	581	98	99
LAC2	*A. thaliana*	Laccase 2	2:12,524,826–12,527,803	573	98	99
LAC5	*A. thaliana*	Laccase 5	2:16,857,958–16,860,779	580	98	99

### Measurements of lignin content, LAC and POD activity in maize ears at different growth stages

**Fig. 6:**
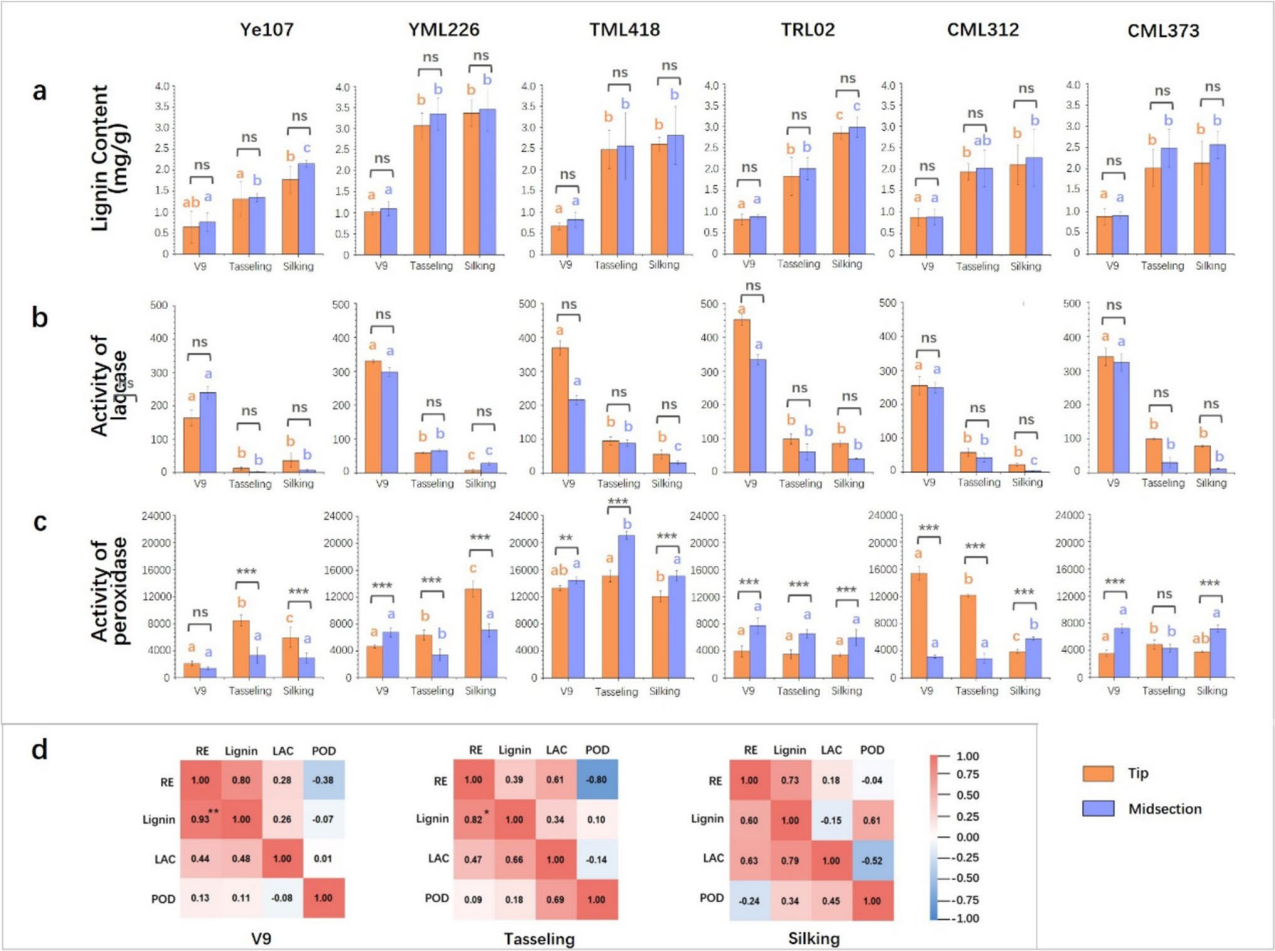
Lignin content, LAC activity and POD activity of six parental lines used in the construction of MPP at three different growth stages. From left to right are Ye107, YML226, TML418, TRL02, CML312 and CML373, respectively. Orange bars represent the tissue from the tip of the cob and purple bars represent the midsection of the cob. **a** Lignin content, **b** LAC activity and **c** POD activity in the tip and midsection tissues of six parental lines at the V9, tasseling, and silking stages. The orange and purple lowercase letters above the corresponding columns represent the significance level at *P* < 0.001 for the tip and midsection tissues at the three sampling stages. The symbols ns, **, and *** indicate no significance, significance at *P* < 0.005, and significance at *P* < 0.001, respectively, between the tip and the midsection tissues. **d** Correlation analysis between RE of *Zm00001d042906*, lignin content, activity of LAC and POD. The data above the diagonal report correlation coefficients for tip section of ears, while data under the diagonal report correlation coefficients for the midsection of ears. * and ** indicate a significance at *P* < 0.05 and *P* < 0.01, respectively

To determine whether *Zm00001d042906* is involved in lignin synthesis in maize cob, we measured lignin content, LAC, and POD activity from samples collected following the same procedure as that for qRT-PCR. The results revealed that at all stages, the lowest lignin content was observed in Ye107, whereas the highest lignin content was observed in YML226 (Fig. 6a, Supplementary Table 5). Across all parental lines, the lignin content was consistently slightly higher in the midsection tissues than in the tip part during the same sampling period, although the differences were not significant. Simultaneously, as ear growth progressed, lignin content in the same section steadily increased in all parental lines (Fig. 6a, Supplementary Table 5). This result indicates that the apical meristematic tissues at the tip of the ear initially have a lower lignin content, but as the cob forms, there is a gradual increase in lignin accumulation, contributing to the toughening of the cob. The results of LAC activity measurements revealed that, at the V9 stage, significantly high laccase activity was observed in all parental lines (Fig. 6b, Supplementary Table 5). The LAC activity in the tip tissues was generally higher than that in the midsection of the cob, except for Ye107. In TML418 and TRL02, the differences between the tip and midsection were large, but not statistically significant (Fig. 6b). This could also be attributed to the relatively small sample size. This finding suggested that LAC activity is the most robust in the apical meristem of the ear tip during the V9 stage. However, as cell differentiation and cob growth progressed, laccase activity in the cells rapidly decreased (Fig. 6b, Supplementary Table 5). Given that POD is also the key enzyme in the final stage of the plant lignin synthesis pathway (Dong and Lin 2020; Gao et al. 2023), we measured POD activity as well from the tip and midsection ears of the six parental lines at the V9, tasseling, and silking stages. The results exhibited diverse trends in POD activities among different parental lines as the growth of the ears progressed. However, in TML418, which had the longest ear, POD activity was higher in all three sampling stages and significantly exceeded the activity in the other parental lines (Fig. 6c, Supplementary Table 5).

Pearson correlation analysis was conducted on the RE of the *Zm00001d042906* gene, lignin content, LAC activities and POD activities at three sampling stages. The results indicated significant correlations between RE and lignin content at 0.93 (*P* < 0.01) and 0.82 (*P* < 0.05) during the V9 and tasseling stages, respectively (Fig. 6d). This suggested the predominant involvement of *Zm00001d042906* in lignin synthesis in the cells at the midsection of the cob.

## Discussion

Tropical and subtropical maize exhibit abundant diversity compared with temperate maize (Grzybowski et al. 2023). Through the utilization a maize MPP constructed with tropical and subtropical maize inbred lines, which display significant variation in EL, we identified a marker, SNP_183573532, and two genes, *Zm00001d042905* and *Zm00001d042906*, located on chromosome 3, encoding laccases. The marker can be considered as a functional marker, and the genes as functional genes regulating maize EL. Previous studies have reported QTLs for EL on Chr3 (Li et al. 2011; Yi et al. 2019; Yang et al. 2020). However, these QTLs did not overlap with the functional regions identified in the present study. This discrepancy could be attributed to the use of tropical and subtropical maize inbred lines, which harbor more favorable variations for EL than temperate maize germplasms. While assessing the broad-sense heritability of MPP, we observed that the EL of maize was predominantly influenced by genetic factors. Therefore, we hypothesize that incorporating tropical and subtropical germplasms into breeding programs to enhance EL in temperate germplasms is feasible due to the beneficial variations carried by tropical and subtropical maize.

Among the six parental lines used in this study, TML418 exhibited the longest EL after maturity. However, the RE of *Zm00001d042906* in TML418 was not high compared to in other long-ear parental lines. Gene expression is influenced by several factors, including genomic suppressors (Bautista et al. 2021), cis-regulatory elements (Yu et al. 2023), the presence of SNPs (Ymer et al. 2002), and environmental factors (Napier et al. 2023). In TML418, the low RE of *Zm00001d042906* may be attributed to SNPs that alter amino acids in the conserved region of the gene (Fig. 4). However, it is noteworthy that although the RE of *Zm00001d042906* was relatively low, the LAC activity of TML418 was not (Figs. 3b and 6b). This could be due to the LAC activity measured in this study encompassed all types of laccases, rather than just *ZmLac4*. Consequently, it is possible that in the samples, other laccases excluding *ZmLac4*, exhibited high activities. POD and LAC are crucial enzymes involved in polymerizing lignin monomers during the later stages of the lignin synthesis pathway (Hoffmann et al. 2020; Gao et al. 2023), the genes regulating POD activity in TML418 may also contribute to ear elongation (Fig. 6c). This finding highlights the complexity of quantitative trait regulation (Holland 2007). The long-ear phenotype of TML418 may result from the combined action of several EL-related genes. In addition, although the RE of *Zm00001d042906* was relatively low in TML418, the GG haplotype at SNP_183573532 appeared to be effective in selecting long-ear maize.

LACs were first reported in lacquer tree (*Rhus vernicifera*) by Yoshida 1883). They belong to a family of multicopper oxidases, serving as multifunctional biocatalysts involved in plant responses to environmental stress (Liang et al. 2006; Yamasaki et al. 2009; Cho et al. 2014; Swetha et al. 2018; Janusz et al. 2020). In recent years, numerous studies have highlighted the role of laccases in lignin synthesis. Zhao et al. (2013) observed that simultaneous disruption of *AtLAC11*, *AtLAC4*, and *AtLAC17* in *Arabidopsis* results in a significant reduction in lignification, providing evidence that LACs are involved in the lignification of *Arabidopsis* stems. Li et al. (2020) demonstrated that the overexpression of *PeLAC10* from bamboo increased lignin content in transgenic *Arabidopsis*. Additionally, it regulates genes associated with drought and phenolic acid tolerance. Liu et al. (2021) discovered that *PtrLAC16* plays a crucial catalytic role in the polymerization of lignin monomers in the cell walls of poplar xylem, particularly in the polymerization of sinapyl lignin.

By measuring the EL, RE of *Zm00001d042906*, lignin content, and LAC and POD activities in the six parental lines of maize at the three growth stages, it was observed that at the V9 stage, the correlation between EL and RE in the midsection was very low (Fig. 3c). This is because at the initial stage of ear development, the tissues were very small, and the differences in EL among the six parental lines were not apparent. However, the lignin content and RE were significantly correlated at the V9 stage (Fig. 6d), indicating the involvement of *Zm00001d042906* in lignin synthesis. At the tasseling stage, RE in the midsection tissues exhibited a significant positive correlation with EL and lignin content (Figs. 3c and 6d). During the silking stage, RE and EL were significantly correlated in the midsection (Fig. 3c). In summary, we hypothesized that *Zm00001d042906* regulates maize EL by participating in lignin synthesis in cells from the midsection of the maize cob. It is well known that lignin content is high in maize cobs. Thus, *Zm00001d042906* may play a vital role in lignin synthesis in the cob cells. When the expression of *Zm00001d042906* was enhanced, lignin content increased, promoting the development and elongation of cells, thereby contributing to the longer EL of maize.

In the present study, some parental lines were selected from the hybrids (Yin et al. 2022; Jiang et al. 2023). For instance, Ye107 and TRL02 were derived from the US hybrid, and TML418, with the longest ear, was derived from the Monsanto hybrid in Thailand (Table 1). Therefore, the candidate gene *Zm00001d042906*, identified from MPP, is likely to have an important value in maize production. In particular, for baby corn that is harvested at the silking stage, *Zm00001d042906* may play a role in elongating maize cobs to increase yield.

In conclusion, this study identified SNP_183573532 and a functional gene, *Zm00001d042906*, on chromosome 3 from tropical and subtropical maize germplasms significantly associated with maize EL. The novel molecular marker and candidate laccase gene identified in this study hold promise for assisting in maize breeding programs by facilitating the selection of maize varieties with elongated ears.

### Supplementary Information

Below is the link to the electronic supplementary material.

Supplementary file1 (XLSX 29 KB)

## Data Availability

The raw data for genotyped individuals of this study are available under NCBI BioProject PRJNA1011459 (https://dataview.ncbi.nlm.nih.gov/?search=SUB13804196).
